# Helicobacter pylori Infection as a Cause of Dyspepsia in the Pakistani Population: An Experience From a Large Tertiary Care Center

**DOI:** 10.7759/cureus.76776

**Published:** 2025-01-02

**Authors:** Saleem Shahzad, Khaild Ahmed Tareen, Ali Hyder, Imran Ahmed, Marium Kauser Siddiqui, Samra Iqbal, Raja Taha Yaseen Khan

**Affiliations:** 1 Hepatogastroenterology, Jinnah Postgraduate Medical Centre, Karachi, PAK; 2 Gastroenterology, Shaikh Khalifa Bin Zayed Al Nahyan Hospital, Quetta, PAK; 3 Gastroenterology, Chandka Medical College, Shaheed Mohtarma Benazir Bhutto Medical University, Larkana, PAK; 4 General Internal Medicine, Princess Royal University Hospital, King’s College Hospital NHS Foundation Trust, London, GBR; 5 Medicine, Dow University of Health Sciences, Karachi, PAK; 6 Hepatogastroenterology, Sindh Institute of Urology and Transplantation, Karachi, PAK

**Keywords:** dyspepsia, gastric biopsy, helicobacter pylori, pakistan, socioeconomic factors

## Abstract

Background

Dyspepsia is one of the most common worldwide gastrointestinal disorders, more prevalent in developing countries like Pakistan. One of the most common causes of dyspepsia is *Helicobacter pylori*. Therefore, this study aimed to determine the frequency of *H. pylori* infection among dyspeptic patients presenting to a tertiary care hospital in Karachi, Pakistan.

Methodology

This cross-sectional study was conducted from January 2021 to March 2022 at Medical Ward VII, Jinnah Postgraduate Medical Center, Karachi. In this study, 370 patients aged 18 years or higher of either gender presenting with symptoms of dyspepsia for more than four weeks were recruited. Patients who received antibiotics within the last four weeks and those with suspected gastric malignancies or liver disease were excluded from the study. Clinical assessment, upper gastrointestinal endoscopy, and histopathological examination of gastric biopsies were performed. Statistical analysis was done using SPSS version 25 (IBM Corp., Armonk, NY, USA), and p-values <0.05 were considered statistically significant.

Results

*H. pylori* infection was diagnosed in 64.8% of patients presenting with dyspepsia. Dominant dyspeptic symptoms included upper abdominal pain in 92.4% of the cases and heartburn in 82.2%. Significant associations with *H. pylori* infection included male gender (p = 0.03), low socioeconomic status (p ≤ 0.01), consumption of outside food (p = 0.012), low hemoglobin levels (p = 0.02), low lymphocyte count (p = 0.017), and decreased lymphocyte count (p = 0.039).

Conclusions

*H. pylori* infection is a very common cause of dyspepsia in Pakistan, and much needs to be done at the public health level to improve hygiene and dietary practices. Longitudinal outcomes, antibiotic resistance, and treatment efficacy are further areas that need to be researched to define an optimal management strategy.

## Introduction

Dyspepsia is one of the most prevalent gastrointestinal complaints, typically defined as persistent or recurrent discomfort or pain in the upper abdomen, often accompanied by symptoms such as bloating, nausea, early satiety, and heartburn [1]. It affects 20-40% of the population worldwide, although the prevalence rate varies geographically and with different socioeconomic environments [2-4]. Dyspepsia has been an important public health issue in Pakistan because it influences quality of life and healthcare costs.

Dyspepsia is caused by several well-known factors, of which *Helicobacter pylori* infection plays a major role [5]. Discovered for the first time in 1982 by Barry Marshall and Robin Warren, *H. pylori *is a Gram-negative, spiral-shaped bacterium with the ability to colonize gastric mucosa [6,7]. It induces chronic gastritis and has been associated with a range of gastroduodenal pathologies, including peptic ulcer disease, gastric cancer, and non-ulcer dyspepsia [8].

The infection rate of *H. pylori* varies from region to region depending upon various factors, including hygiene, sanitation, socioeconomic conditions, and cultural background. In the developed world, the infection rate ranges from 20% to 50%, while in the developing parts of the world, the infection rate is above 70% [9-12]. In South Asia, local studies have reported prevalence rates as high as 60-80% [13]. Liaqat Memon et al. reported *H. pylori* in 47% of patients presenting with dyspepsia in the Pakistani population [14].

*H. pylori* is spread mainly by the fecal-oral or oral-oral route, usually during childhood. The bacterium destroys the gastric mucosal barrier through the release of huge amounts of cytotoxins, including vacuolating cytotoxin A (VacA), and cytotoxin-associated gene A (CagA), thereby producing chronic inflammation of gastritis and at times peptic ulceration. *H. pylori* promotes several pathophysiological mechanisms in dyspeptic patients, such as an increase in acid secretion, impaired gastric motility, and gut-brain signal alteration [15].

Although *H. pylori* is known for being associated with dyspepsia, few data have been gathered concerning this infection as a cause of dyspepsia in Pakistani patients. Infection caused by *H. pylori* is treatable; hence, assessing its prevalence will lead to optimization of patient management. Understanding the burden of *H. pylori* infection can help in taking preventive measures, such as enhancing hygiene and sanitation. Therefore, this study aimed to determine the frequency of *H. pylori* infection among dyspeptic patients presenting to the outpatient department of Medical Ward VII, Jinnah Sindh Medical University.

## Materials and methods

This cross-sectional study was conducted at the outpatient clinic of Medical Ward VII, Jinnah Sindh Medical University, Karachi from January 1, 2021, to March 31, 2022, after obtaining approval from the Institutional Review Board-Jinnah Postgraduate Medical Center (IRB-JPMC) (approval number: 7784) and taking the informed consent from all patients. All patients of either gender and aged 18 years or more and presenting with symptoms of dyspepsia for more than four weeks were enrolled in the study. Patients with a history of recent use of any antibiotics or proton pump inhibitors or those with a history of gastric malignancies or previous gastric surgeries; those with a history of liver disease or portal gastropathy; those with a history of antiplatelet medications or non-steroidal anti-inflammatory drugs; pregnant women; and lactating mothers were excluded from the study.

Sample size estimations were done assuming that the prevalence of H. Pylori infection among dyspeptic patients would be 60% with a 95% confidence interval and margin of error of 5%. Thus, using Cochran's formula, an estimated sample size calculated was determined to be 370 patients.

Data collection procedure

Consecutive patients meeting the inclusion criteria were enrolled from the outpatient department. All demographic and clinical data, such as age, sex, socioeconomic status, dietary history, and duration of symptoms, were noted on a predesigned proforma. *H. pylori* was diagnosed by performing an upper gastrointestinal endoscopy and biopsies from the body and antrum of the stomach in each patient. For the detection of *H. pylori*, biopsy specimens were immersed in formalin and sent for histopathological examination by an expert histopathologist with more than 20 years of experience in gastrointestinal and infectious diseases.

Data analysis procedure

The statistical analysis was performed using SPSS version 25.0 (IBM Corp., Armonk, NY, USA). For categorical data, frequencies and percentages were used while continuous variables were expressed as mean and standard deviation. The outcome was observed in terms of the presence or absence of *H. pylori* infection. Comparative analysis was performed using the chi-square test for categorical variables and the Student’s t-test for continuous variables. P-values <0.05 were considered significant.

## Results

A total of 370 patients with dyspepsia were enrolled in the study. Of them, 207 (55.9%) patients were females, and 163 (44.1%) patients were males. The mean age was 42.6 ± 12.3 years. Low socioeconomic status was noted in 240 (64.8%) patients. The most common presenting complaint was upper abdominal pain in 342 (92.4%) patients, followed by heartburn in 304 (82.2%), bloating in 277 (74.5%), early satiety in 144 (38.9%), and unintentional weight loss in 74 (20%) patients. Consumption of meals from outside was noted in 220 (59.5%) patients. On endoscopy and histopathology, *H. pylori* was noted in 240 (64.8%) patients (Table 1).

**Table 1:** Baseline characteristics of the study population (n = 370). *H. pylori* = *Helicobacter pylori*; TLC = total leucocyte count

Study population		n (%)
Gender	Males	163 (44.1)
	Females	207 (55.9)
Low socioeconomic status	Yes	240 (64.8)
	No	130 (35.2)
Symptoms of dyspepsia at baseline	Epigastric pain	342 (92.4)
	Bloating	277 (74.5)
	Weight loss	74 (20)
	Early satiety	144 (38.9)
	Heartburn	304 (82.2)
Consumption of meals from outside	Yes	220 (59.5)
	No	150 (40.5)
*H. pylori* on histology	Present	240 (64.8)
	Absent	130 (35.2)
Mean age (years ± SD)		42.6 ± 12.3
Hemoglobin (g/dL)		10.4 ± 2.7
TLC (×10^9^/L)		6.9 ± 1.4
Absolute neutrophil count (×10^9^/L)		74.8 ± 3.1
Absolute lymphocyte count (×10^9^/L)		14.9 ± 2.9
Platelet (×10^9^/L)		341 ± 78

On comparative analysis, male gender (p = 0.03), low socioeconomic status (p ≤ 0.01), consumption of outside food (p = 0.012), decreased hemoglobin levels (p = 0.02), increased platelet count (p ≤ 0.01), decreased total leucocyte count (p = 0.041) and lymphocyte count (p = 0.017), and increased neutrophil count (p = 0.039) were significantly associated with *H. pylori* infection (Table 2).

**Table 2:** Factors associated with H. pylori infection. *H. pylori* = *Helicobacter pylori*; TLC = total leucocyte count

Variable		H. pylori on gastric biopsy		P-value
		Present (n = 240), n (%)	Absent (n = 130), n (%)	
Gender	Male	144 (60)	19 (14.7)	0.003
	Female	96 (40)	111 (85.3)	
Low socioeconomic status	Yes	178 (74.2)	62 (47.7)	≤0.001
	No	62 (25.8)	68 (52.3)	
Consumption of food from outside	Yes	174 (72.5)	46 (35.4)	0.012
	No	66 (27.5)	84 (64.6)	
Mean age (years ± SD)		43.0 ± 12.1	41.9 ± 13.5	0.45
Hemoglobin (g/dL)		9.8 ± 3.1	10.7 ± 2.4	0.02
TLC (×10^9^/L)		6.4 ± 3.2	7.8 ± 1.4	0.041
Absolute neutrophil count (×10^9^/L)		71.4 ± 3.7	63.1 ± 5.4	0.039
Absolute lymphocyte count (×10^9^/L)		13.4 ± 1.7	16.1 ± 2.8	0.017
Platelet (×10^9^/L)		384 ± 151	227 ± 98	≤0.001

## Discussion

The results of this study showed the high prevalence of *H. pylori* infection in dyspeptic patients in Pakistan, especially at a tertiary care center. The prevalence of *H. pylori* infection in this study was 64.8%, which agrees with reports from developing countries, where prevalence frequently exceeds 60%.

A few studies conducted in developing South Asian neighboring countries also reported prevalence ranges between 60% and 80% due to similar socioeconomic and poor hygienic conditions prevailing in these regions. A study by Liaqat Memon et al. reported a prevalence of 47% among dyspeptic patients in Pakistan, which is relatively lower compared to the present findings [14]. The variation perhaps relates to differences in study designs, methods adopted for diagnosis, and different populations studied.

The prevalence is very different between industrialized and developing countries. In the developed world, the prevalence ranges between 20% and 50% due to effective sanitation and healthcare infrastructure in place. Several studies from the United States showed a prevalence of 25-35% of *H. pylori* among dyspeptic patients, strongly pointing toward the role of socioeconomic factors in the dispersal of *H. pylori* [16,17]. This is consistent with the higher prevalence of *H. pylori* seen in low socioeconomic countries like Pakistan, where factors such as poor sanitation, high population density, and lack of clean water supplies are important risk factors for *H. pylori* infection [18].

The most frequent symptom among patients was upper abdominal pain (92.4%), followed by heartburn (82.2%), and bloating (74.5%). These findings are in agreement with other studies reporting similar symptomatology in *H. pylori*-associated dyspepsia [19]. The strong association of *H. pylori* with low socioeconomic status, male gender, and dietary habits such as the consumption of outside food is in agreement with studies conducted worldwide [20].

Of note, laboratory findings such as low hemoglobin levels, increased platelet count, and changes in leucocyte profiles showed significant association with *H. pylori* infection in this study. Such hematological changes are likely to be secondary to chronic inflammation and iron deficiency caused by the bacterium. Previous studies have similarly reported anemia and thrombocytosis as indirect markers of chronic *H. pylori* infection, emphasizing the systemic impact of this pathogen beyond the gastrointestinal tract [21].

Although this study gives valuable input, some limitations need to be considered. First, as this is a single-center study, its findings cannot be generalized to the general population. Second, it is a cross-sectional design, and therefore causal relations cannot be established. Future research should investigate longitudinal outcomes of *H. pylori*-associated dyspepsia, the effectiveness of different eradication regimes, and the impact of antibiotic resistance on treatment failure.

## Conclusions

This study has highlighted the higher burden of *H. pylori* infection among dyspeptic patients in Pakistan, similar to most other developing countries. In fact, the findings emphasize the importance of socioeconomic factors, diet-related risk factors, along with various hematological parameters playing significant roles in the prevalence and presentation pattern of *H. pylori*-infected dyspepsia. These include poor sanitation, high population density, and inadequate access to clean water, all of which contribute to its high prevalence and the need for reinforcing public health hygiene and improved living conditions. The development of longitudinal outcomes, assessment of the efficacy of eradication regimens, and emerging challenges such as antibiotic resistance are some areas that should be studied in future research.
